# Risk of Incident Non-Valvular Atrial Fibrillation after Dialysis-Requiring Acute Kidney Injury

**DOI:** 10.3390/jcm7090248

**Published:** 2018-08-29

**Authors:** Chih-Chung Shiao, Wei-Chih Kan, Jian-Jhong Wang, Yu-Feng Lin, Likwang Chen, Eric Chueh, Ya-Ting Huang, Wen-Po Chiang, Li-Jung Tseng, Chih-Hsien Wang, Vin-Cent Wu

**Affiliations:** 1 Division of Nephrology, Department of Internal Medicine, Saint Mary’s Hospital Luodong, Loudong 265, Yilan, Taiwan; chungyy2001@yahoo.com.tw; 2 Saint Mary’s Junior College of Medicine, Nursing and Management, Sanxing Township, Yilan County 266, Taiwan; 3 Division of Nephrology, Department of Internal medicine, Chi Mei Medical Center, Yongkang District, Tainan City 710, Taiwan; rockiekan@ntu.edu.tw; 4 Department of Biological Science and Technology, Chung Hwa University of Medical Technology, Rende District, Tainan City 717, Taiwan; 5 Division of Nephrology, Department of Internal Medicine, Chi Mei Medical Center, Liouying, Tainan City 736, Taiwan; win7a@yahoo.com.tw; 6 Graduate Institute of Clinical Medicine, National Taiwan University College of Medicine, Taipei 106, Taiwan; dr.yufenglin@gmail.com; 7 Division of Nephrology, Department of Internal Medicine, National Taiwan University Hospital, Taipei 100, Taiwan; 8 Division of Hospital Medicine, Department of Internal Medicine, National Taiwan University Hospital, Taipei 100, Taiwan; 9 Institute of Population Health Sciences, National Health Research Institutes, Zhunan, Miaoli County 350, Taiwan; likwang@nhri.org.tw; 10 Case Western Reserve University, No. 10900 Euclid Ave., Cleveland, OH 44106, USA; ericc0109@gmail.com; 11 Department of Nursing, Saint Mary’s Hospital Luodong, Loudong 265, Yilan, Taiwan; frankie7451@gmail.com; 12 Department of Surgery, National Taiwan University Hospital, Taipei 100, Taiwan; aillen1111@yahoo.com.tw (W.-P.C.); ntuhtlj@gmail.com (L.-J.T.); wchemail@gmail.com (C.-H.W.); 13 NSARF, National Taiwan University Study Group on Acute Renal Failure

**Keywords:** acute kidney injury, adverse cardiovascular events, atrial fibrillation, dialysis

## Abstract

The influence of acute kidney injury (AKI) on subsequent incident atrial fibrillation (AF) has not yet been fully addressed. This retrospective nationwide cohort study was conducted using Taiwan’s National Health Insurance Research Database from 1 January 2000 to 31 December 2010. A total of 41,463 patients without a previous AF, mitral valve disease, and hyperthyroidism who developed de novo dialysis-requiring AKI (AKI-D) during their index hospitalization were enrolled. After propensity score matching, “non-recovery group” (*n* = 2895), “AKI-recovery group” (*n* = 2895) and “non-AKI group” (control group, *n* = 5790) were categorized. Within a follow-up period of 6.52 ± 3.88 years (median, 6.87 years), we found that the adjusted risks for subsequent incident AF were increased in both AKI-recovery group (adjusted hazard ratio (aHR) = 1.30; 95% confidence intervals (CI), 1.07–1.58; *p* ≤ 0.01) and non-recovery group (aHR = 1.62; 95% CI, 1.36–1.94) compared to the non-AKI group. Furthermore, the development of AF carried elevated risks for major adverse cardiac events (aHR = 2.11; 95% CI, 1.83–2.43), ischemic stroke (aHR = 1.33; 95% CI, 1.19–1.49), and all stroke (aHR = 1.28; 95% CI, 1.15–1.43). (all *p* ≤ 0.001, except otherwise expressed) The authors concluded that AKI-D, even in those who withdrew from temporary dialysis, independently increases the subsequent risk of de novo AF.

## 1. Introduction

Atrial fibrillation (AF) is the most common sustained cardiac arrhythmia with an increasing trend of prevalence following widespread population aging [1]. It is estimated that AF currently affects around 2.3 million adults in the United States, and the number of affected people is projected to increase to 5.6–15.9 million by 2050 [1]. AF has a significant effect on cardiovascular events including stroke, peripheral embolization, and associated morbidities and mortalities [1]. The existence of AF is independently associated with the risk of the development and severity of acute kidney injury (AKI) in both surgical and medical settings [2].

Recent studies suggest that AKI episodes are associated with a higher risk of developing cardiovascular events and overall mortality [3,4,5]. Nevertheless, the influence of AKI on subsequent incident AF has not been fully addressed in previous research. Most investigations only evaluate the concurrent occurrence of AKI and AF in a limited number of patients undergoing cardiac surgeries within a relatively short follow-up period [6,7]. In these studies, the development of postoperative AKI was found as an independent factor associated with the new-onset postoperative AF [6,7]. In line with these interpretations, new-onset AF after AKI in a nationwide survey for an extended followed-up period is necessary [7,8,9,10]. In particular, the National Institute for Health and Clinical Excellence (NICE) guideline raises an ultimate critical point: if the development of AF occurs, the assessment to evaluate the risk of having a stroke is necessary. However, little information is available regarding cardiovascular events and outcomes after new-onset AF in this subset.

A proper understanding of the risk factors associated with kidney disease and AF development may allow primary care physicians to initiate preventive strategies and thereby potentially decrease the risk of AF. Thus, we conducted this study aiming to test the hypothesis that the occurrence of dialysis-requiring AKI (AKI-D), as well as the “recovery pattern” of the AKI, would increase the subsequent risk of subsequent AF and result in worse cardiovascular injury.

## 2. Materials and Methods

### 2.1. Data Source

This retrospective population-based cohort study was conducted using the data of Taiwan’s National Health Insurance Research Database (NHIRD) in the period from 1 January 2000 to 3 December 2010. The NHIRD is an encrypted database released by the National Health Research Institutes (NHRI) for research purposes. The NHIRD includes all information on outpatient visits, hospital admissions, prescriptions, interventional procedures, disease profiles, and vital status of the National Health Insurance (NHI) program which provides comprehensive medical care covering more than 99% of the country’s population of 23 million people. The baseline comorbidities were identified from at least three outpatient visits or one inpatient claim within one year preceding the index hospitalization with the first dialysis. This identification method has been well validated with adequate predictive power [3]. The Charlson Comorbidity Index (CCI) was calculated by weighting baseline comorbidities.

For confidentiality purposes, identification numbers were encrypted before being released for research, but the uniqueness of the encrypted identification is retained to ensure valid internal linkage. The study protocol conformed to the ethical guidelines of the 1975 Declaration of Helsinki. The study was approved by the institutional review board of the National Taiwan University Hospital (201212021RINC), and informed consent was waived since all personal data were de-identified in the database to protect privacy.

### 2.2. Study Cohort and Design

This study included patients aged 18 to 100 years without a history of AF, mitral valve disease, and hyperthyroidism for at least one year preceding study enrollment, and who developed de novo AKI-D during their index hospitalization and survived to at least 30 days after discharge.

Disease diagnoses were classified according to the International Classification of Diseases, Ninth Revision, Clinical Modification (ICD-9-CM). AKI-D was defined by ICD-9 codes for AKI (584.3, 634.3, 635.3, 636.3, 637.3, 638.3, 639.3, 669.3, or 958.5) along with procedure codes for acute dialysis. CKD was defined by ICD-9 codes for CKD (580, 580.x, 584.x, 586, 399.5). Advanced CKD was defined as CKD patients with concomitant erythropoiesis-stimulating agents [3]. The diagnosis accuracy of AKI and chronic kidney disease (CKD) by ICD-9-CM, as well as the definition of advanced CKD, were detailed in Supplementary File 1.

Renal function recovery was defined by withdrawal from dialysis before the 31st day after discharge. The patients who had successfully withdrawn from dialysis within hospitalization or within the 30-day period after hospital discharge were categorized into “AKI-recovery” group, while those had not withdrawn from dialysis within the 30-day period were categorized in “non-recovery” group. In an attempt to make a less biased comparison, we further constructed a control group which contained patients without AKI and who survived to discharge from the remaining hospitalized patients (non-AKI group).

### 2.3. Research Variables

The demographic data including age, gender, monthly income, hospital levels, baseline comorbidities, CHA2DS2-VASc scores before discharge, the frequency of outpatient visits and medications within one year following discharge, and the patients’ outcomes were identified and analyzed.

### 2.4. Outcome Variables

Our primary outcome was de novo AF development (ICD-9-CM code 427.31) after hospital discharge. To ensure the accuracy of the AF identification, the diagnosis of AF needed to be confirmed by a doctor, or doctors, and recorded in the diagnosis list of the chart more than twice in outpatient visits, or recorded in the discharge diagnosis list more than once in an inpatient setting. The diagnostic accuracy of AF based on the ICD-9-CM codes has been previously validated [11,12,13]. The secondary outcomes included major adverse cardiac event (MACE), ischemic stroke (ICD-9-CM code 433.x, 434.x, or 436), and hemorrhage stroke (ICD-9-CM code 431 or 432). MACE was defined as coronary artery disease-related death, nonfatal myocardial infarction, angina, and revascularizations.

We evaluated the influences of AKI-D on the risk of subsequent incident AF in the whole study population as well as in different subgroups. Furthermore, besides testing the association between AKI-D and these outcomes, we also evaluated the association between AF and the above-mentioned secondary outcomes. All enrolled patients were followed from the date of index hospital discharge to the first diagnosed outcomes and were censored at death unrelated to coronary artery disease, or at the end of the study (31 December 2010).

### 2.5. Statistical Analysis

Statistical analyses were performed using SAS 9.4 (SAS Institute, Cary, NC, USA). A two-sided *p*-value of ≤ 0.05 was considered statistically significant. Continuous variables were presented as the mean ± standard deviation (SD), and categorical variables were described as counts (percentages). The propensity scores were determined by multivariate logistic regression analysis. The Cox proportional hazard model was applied to examine the effect of non-recovery and AKI-recovery groups on subsequent AF development, as well as the influence of AF on other major adverse events. Because withdrawal from dialysis is likely to adhere to the condition of acute kidney disease, advanced CKD was identified to be a time-varying covariate [3,14].

By using the propensity score matching method, we proposed three matched groups (non-recovery group: AKI-recovery group: non-AKI group) on a 1:1:2 ratio. The propensity scores, containing the baseline characteristics and risk factors listed in Table 1, were calculated by multivariate logistic regression.

Several demographic factors were adjusted in the hazard models to evaluate the impact of AKI on AF, as well as the influence of incident AF to other adverse events. Variable selection was performed using stepwise multiple regression methods, with both *p*-to-enter and *p*-to-leave equal to 0.15. Final results of multivariate analyses were presented by hazard ratio (HR) and adjusted HR (aHR) with 95% confidence interval (CI). To exclude the confounding effect of subsequent impaired renal function and the influence of chronic dialysis, we additionally took “advanced CKD” as a “time-varying covariate” in the Cox proportional hazard model determining the adjusted risk for subsequent AF.

## 3. Results

### 3.1. Characteristics of the Three Groups

**Figure 1:**
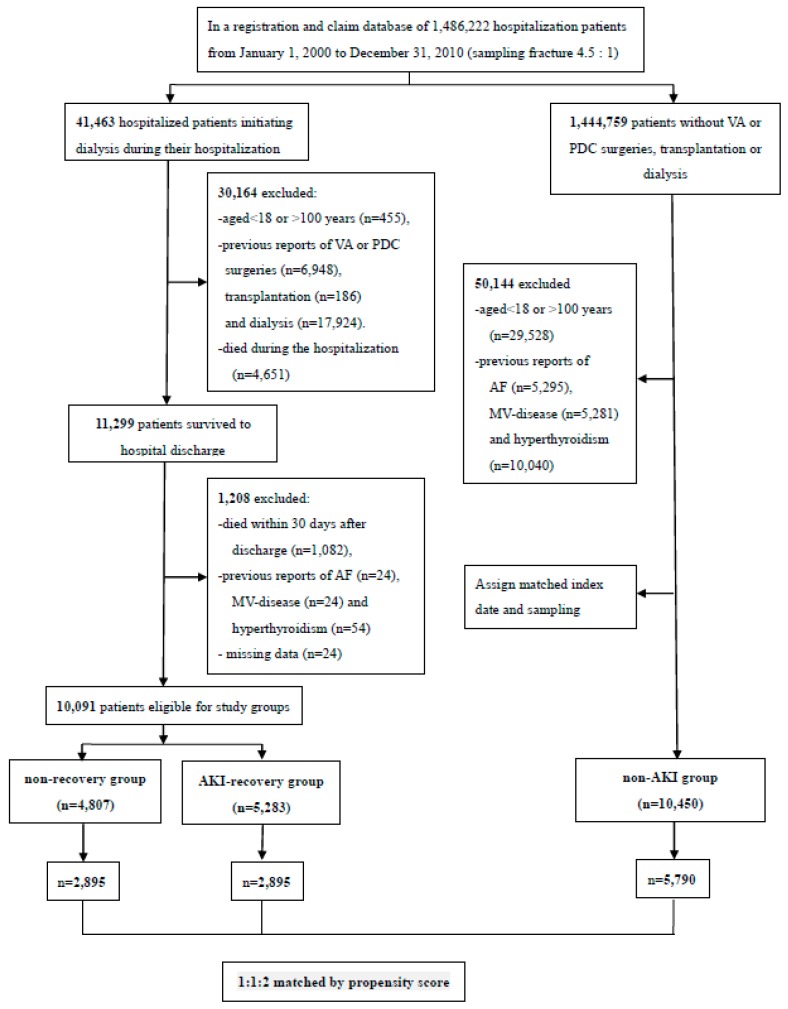
Study flow diagram. Abbreviations: AF, atrial fibrillation; MV, mitral valve; PDC, peritoneal dialysis catheter; VA, vascular access.

From the period of 1 January 2000, to 31 December 2010, a total of 10,091 AKI-D patients were eligible for inclusion. Among them, 5284 patients withdrew from dialysis for at least 30 days, but 4807 patients did not. After the exclusion and sampling processes, a total of 10,450 patients without AKI and who survived to hospital discharge were enrolled as control group. After propensity score matching on a 1:1:2 ratio, we categorized these patients into non-recovery (*n* = 2895), AKI-recovery (*n* = 2895) and non-AKI (*n* = 5790) groups (Figure 1).

The follow-up period of the whole post-matching cohort was 6.38 ± 3.83 years (median, 6.68 years; range, 0.02–10.99 years). Among the three groups, the non-AKI group was the oldest. While the gender, socioeconomic status, baseline comorbidities, as well as outpatient follow-up visits and medication status were not statistically different among the three groups (Table 1). All primary outcome (AF, *p* = 0.002) and secondary outcomes (MACE, ischemic stroke, hemorrhage stroke and all stroke, all *p* ≤ 0.001) were of statistical significance among the three groups (Table 2).

### 3.2. Risk of Incident Atrial Fibrillation

The occurrence time of AF since discharge was 3.24 ± 2.94 years (median, 0.24 years; range, 0.02–10.94 years). The incidence rates of AF were 0.94%, 1.14%, and 1.34% before propensity score matching in “non-AKI group”, “AKI-recovery group”, and “non-recovery group”, respectively.

**Figure 2:**
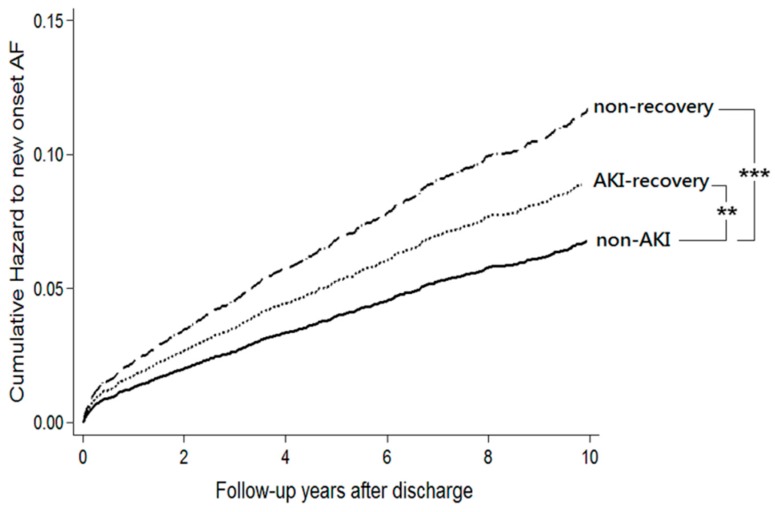
Cumulative incidences of atrial fibrillation among the three groups. Note: The analysis was performed using the Cox proportional hazard method with adjustment to the Charlson Comorbidity Index, age, gender and advanced chronic kidney disease (time-varying covariate). *** denotes *p* < 0.001, ** denotes *p* < 0.01. Abbreviations: AF, atrial fibrillation; AKI, acute kidney injury.

After propensity score matching, the adjusted risks for subsequent de novo AF development were significantly higher in both the “non-recovery group” (aHR = 1.62; 95% CI, 1.36–1.94; *p* ≤ 0.001) and the “AKI-recovery group” (aHR = 1.30; 95% CI, 1.07–1.58; *p* ≤ 0.01) compared to the “Non-AKI group” (Table 3 and Figure 2). When compared with “non-recovery group”, the “AKI-recovery group” had a significantly lower risk of subsequent AF (aHR = 0.80; 95% CI, 0.616–0.933; *p* ≤ 0.01).

### 3.3. Risk of Incident Atrial Fibrillation in Subgroups

**Figure 3:**
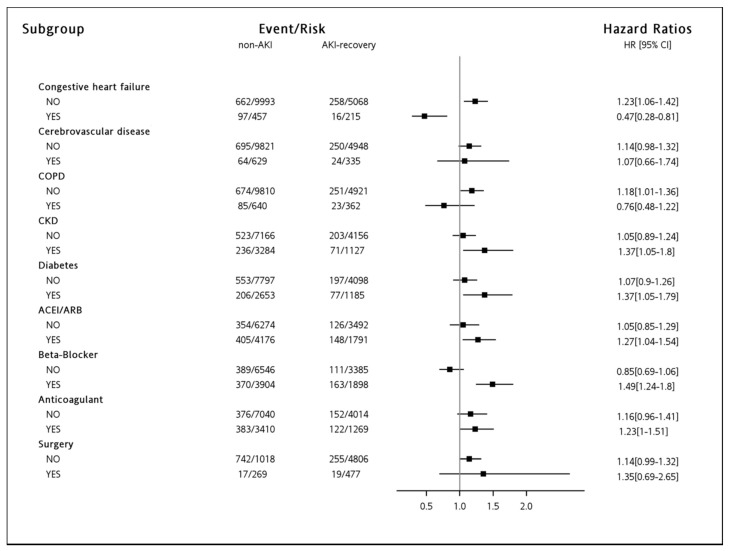
Subgroup comparisons for the risk of atrial fibrillation. Note: The forest plot for the comparison of AKI-recovery group versus non-AKI group was drawn using the before-matching population with adjustment to the Charlson Comorbidity Index, age, and gender. Abbreviations: ACEI, angiotensin-converting enzyme inhibitor; ARB, angiotensin receptor blocker; AF, atrial fibrillation; AKI, acute kidney injury; CKD, chronic kidney disease; COPD, chronic obstructive pulmonary disease.

In the subgroup comparison, we found that “AKI-recovery group” has a significantly higher risk of incident AF than the “non-AKI group” in some subgroups. These subgroups included the patients without congestive heart failure (CHF) (aHR = 1.23; 95% CI, 1.06–1.42) and chronic obstructive pulmonary disease (aHR = 1.18; 95% CI, 1.01–1.36), those with CKD (aHR = 1.37; 95% CI, 1.05–1.80) and diabetes mellitus (aHR = 1.37; 95% CI, 1.05–1.79), as well as those receiving angiotensin-converting-enzyme inhibitor (ACEI)/angiotensin receptor blocker (ARB) (aHR = 1.27; 95% CI, 1.04–1.54) and beta-blocker (aHR = 1.49; 95% CI, 1.24–1.80). Additionally, the subgroups who received anticoagulants (aHR = 1.23; 95% CI, 1.00–1.51) had marginally significant increased risks of AF (Figure 3).

### 3.4. Risk of Major Adverse Events between Patients with and without Incident Atrial Fibrillation

After propensity score matching, the patients with de novo AF augmented risks for MACE (aHR = 2.11; 95% CI, 1.83–2.43), ischemic stroke (aHR = 1.33; 95% CI, 1.19–1.49) and all stroke (aHR = 1.28; 95% CI, 1.15–1.43) (all *p* ≤ 0.001) (Table 4).

## 4. Discussion

This study is the first to demonstrate a long-term association between AKI and the subsequent incident AF in critically ill patients using a large nationwide cohort. We found that the experience of severe AKI necessitating dialysis, even in the patients who only required temporary dialysis, was associated with increased risk of subsequent incident AF. Moreover, the experience of incident AF further carried a higher risk of MACE, and ischemic stroke in these patients. Since AKI carries an increased risk of coronary events [3], the increased AF following AKI could at least partially explain the elevated probability of MACE after AKI. Since the current study was designed using a selected population set from a nationwide database to evaluate the influence of AKI-D on the incident AF, the incidence rate of AF in this study was not comparable to other investigations [15,16] for an epidemiological purpose.

Most of the previous studies evaluating the association between AF and AKI were designed with a relatively short study period using patients who underwent cardiac surgeries [6,7]. One such study enrolled 446 cardiac surgical patients and found that the development of postoperative AKI was an independent factor associated with new-onset AF [6]. Another prospective study including 2572 cardiac surgical patients disclosed that the occurrence of postoperative AKI carried the 1.7-fold increased risk of developing postoperative AF [7]. In the subgroup comparison of the current study, although insufficient case numbers made the results not statistically significant, we also observed a tendency that patients receiving cardiac surgeries had a higher risk of incident AF than those without (aHR, 1.35 versus 1.14) (Figure 3). Nevertheless, the results from post-cardiac surgical patients probably could not be extensively applied to other patient settings, because the cardiac surgical patient is a special population with a higher risk of AF due to the relevant involvement of heart structurally and electrically [8,17]. Furthermore, the temporal association between AKI and AF was difficult to be clarified by these studies because of a short period of observation [7,9,10]. Compared to the previous studies, the clarified temporal association between in-hospital AKI and new-onset AF after hospital discharge, along with the long-term follow-up period in the current study, provides more strengthened evidence in this field.

In particular, we provide an important outcome estimate: that is, even after being adjusted with progression to subsequent advanced CKD or ESRD, in the worst of circumstances, AKI still independently contributes to subsequent incident AF or all-cause mortality. Therefore, the risk factors of the kidney injury that had engendered an AKI event may persist and eventually lead to future AF without direct causal association to the subsequent CKD.

### 4.1. Acute Kidney Injury and Atrial Fibrillation

An increasing body of evidence has shown that AKI is independently associated with the occurrence of AF [18]. AKI causes “remote organ injury” in the heart by the “classical” acute uremic effect, the inflammatory state and the modulating effect of the underlying morbidities associated with the injured kidneys, as well as the health care dilemma [5].

Several mechanisms are proposed for explaining the association between AKI and the elevated risk of AF: (1) The increased preload because of the AKI-induced salt and water retention at the acute stage could increase the cardiac structural change [19,20,21,22]. (2) The myocardial damage and impaired left ventricular function secondary to the enhanced neutrophil trafficking, endothelial dysfunction, myocyte apoptosis, as well as an increased level of inflammatory cytokines [19,20,21,22]. Additionally, fibroblast growth factor 23 (FGF-23), a biomarker for predicting early AKI presentation and cardiovascular morbidity and mortality [23], is disclosed to have markedly elevated serum level in patients with AKI. Higher FGF-23 levels were also found to be associated with elevated risk of AF development in both patients with and without clinical cardiovascular disease [24]. Thus, the enhanced AF incidence might be attributed to the atrial remodeling related to increased FGF-23 levels [25]. Taken together, these results provide a new perspective on the pathogenesis of sinoatrial dysfunction after AKI and open new avenues for treatment of the disease. (3) The activation of the sympathetic nervous system: previous studies have demonstrated that ischemia-reperfusion injury related AKI would activate a sympathetic reflex [26]. The activating sympathetic activation of the atrium would subsequently cause remodeling of the cardiac autonomic neural tissue and promote further persistence and recurrence of AF [27].

Of note, in the subgroup of “patients without CHF”, the finding that “recovery AKI-D patients had a higher risk of subsequent AF than non-AKI patients” was consistent with the results from our whole study population. This was reasonable when we considered this finding as a result without the confounding the effect of CHF. Nevertheless, diverse observations were disclosed in patients with CHF. In these patients, the indication of dialysis could more likely be “fluid overload”, which was associated with a more favorable prognosis than other indications. On the contrary, the mortality risk might be higher in patients with more severe AKI-D and CHF than those who only had AKI-D. Owing to the lack of the etiology and severity of congestive heart failure, and the indication of dialysis in the database for further analysis, the observation among patients with CHF should be inconclusive (Figure 3).

Additionally, the results among the subgroups of “hypertensive patients taking beta-blocker and ACEI/ARB” were also consistent with the findings from the whole population. These findings could be interpreted as the beneficial effects of the aforementioned anti-hypertensive medications minimizing the other confounding effects of the underlying cardiovascular disease. In contrast, the influences of AKI-D on AF were blunted in the patients not taking beta-blockers and ACEI/ARB. In fact, this subgroup contains two patient groups (patients without hypertension, and hypertensive patients not taking beta-blocker or ACEI/ARB for treatment) with different prognostic characteristics. Without doing further analysis, we could not draw any conclusions regarding this issue (Figure 3).

### 4.2. Major Adverse Events Associated with Atrial Fibrillation

In the current study, incident AF was associated with several major adverse events (Table 4). Similar findings were also demonstrated that the development of AKI-D was associated with increased risk of in-hospital mortality and adverse events among patients with AF [2]. Following our results, patients who have AF and AKI will have a high incidence of cardiovascular events, especially coronary events and ischemic stroke. Distant organ injury, a direct consequence of deleterious systemic effects following AKI, is an important issue for clinical care [5].

The association between AF and abnormal renal function is also an interesting and essential issue to be discussed. Among stable anticoagulated patients with AF, the presence of impaired baseline renal function was reported as an independent risk factor of the occurrence of thrombotic/vascular events, stroke, bleeding, and mortality [28,29]. During the two-year follow-up period, about 21% to 32% of patients had a rapid renal function deterioration (decline of estimated Glomerular filtration rate (eGFR) > 5–10 mL/min/1.73 m^2^) [28,30]. More renal function deterioration in absolute levels (decreases of eGFR ≥ 15–25 mL/min/1.73 m^2^ within two years) or relative percentage (decline of ≥ 25% eGFR) within 2 years was independently associated with the occurrence of stroke or death in these patients [29]. The independent risk factors for the development of severe kidney disease include diabetes, CHF, coronary artery disease and impaired baseline renal function [28]. However, normal or near-normal baseline renal function did not exclude the subsequent development of severe renal impairment over time [28].

Regarding the therapeutic strategy for AF, the old drug “Warfarin” is thought to be associated with higher risk for AKI, which is probably due to a higher risk of glomerular hemorrhage known as anticoagulation-related nephropathy [31]. In a randomized study enrolling 18,113 patients, Bohm et al. [32] found that patients receiving warfarin, as opposed to those receiving direct oral anticoagulants (DOAC), have more rapid eGFR decline after an average follow-up period of 30 months. Patients with poor international normalized ratio control (i.e., time in the therapeutic range <65%) tend to have a faster decline in eGFR. Similarly, Chan et al. [33] found that DOAC users had an overall 21% lower risk of AKI compared with warfarin users among Asians with nonvalvular AF. The beneficial effect was especially disclosed in among patients with eGFR > 60 mL/min/1.73 m^2^.

As to the association between AF and MACE, some information should be addressed. In a prospective cohort of 23,928 participants without coronary artery disease conducted by Soliman et al. [34], AF was associated with around two-fold elevated the risk of myocardial infarction within a 6.9 year (median 4.5 years) follow-up. Consistent findings were found in the study enrolling 4,608 participants by O’Neal et al. [35]. Within the median follow-up period of 12.2 years, 17.3% participants developed myocardial infarction, and AF independently carried 1.7 folds increase the risk of myocardial infarction. The increased cardiovascular risk of AF was further confirmed by a systemic review and meta-analysis including 15 cohort studies. Another work also found that AF is associated with an elevated risk of CHF and all-cause mortality in patients regardless of having coronary artery disease, and additionally with an elevated risk of subsequent myocardial infarction in those without coronary artery disease [36]. Of note, the influences of AF on increasing risk of cardiovascular events were more prominent in women than men and African-Americans than Caucasians [34,35,37].

Despite receiving oral warfarin treatment, patients with AF still have a high rate of cardiovascular events, including fatal/nonfatal myocardial infarction, cardiac revascularization, and cardiovascular death. The independent predictors of cardiovascular events included age, smoking, history of cerebrovascular and cardiac events, metabolic syndrome, CHF, and male gender [38].

The cardiovascular safety of oral anticoagulants has long been debated. By using a systemic review and meta-analysis, Tornyos et al., [39] disclosed that most of the DOACs were of safer than warfarin regarding the risk of subsequent myocardial infarction. A very recent work by Lee et al. [40] further exhibited a favorable effect of DOACs as compared to warfarin in reducing cardiac complications in AF. In the analysis using 31,739 patients, all DOACs were associated with lower risk of myocardial infarction than warfarin.

AKI is now considered a growing global health alert [41]. These findings are noteworthy from the perspective of a clinician caring for an individual with dialysis-requiring AKI. Considerable growth in AKI epidemiology and improvements in post-hospitalization resource utilization [42] allowed us to examine the impact of AKI on AF and identify a large vulnerable population with increased risk of cardiovascular events and all-cause mortality [3,4].

### 4.3. Limitations

Several limitations are worth mentioning. First, the nature of the observational study is subject to bias. Second, the study using administrative data is potentially limited by unmeasured confounding. The epidemiological data, the etiology, and severity of AKI, the indication of dialysis initiation, as well as some known risk factors including alcohol abuse and body mass index, which may further provide meaningful information regarding the association with subsequent AF, are not available in such a nationwide insurance research database. Third, the primary outcome of the current study is “non-valvular AF”. The observations accrued here might not be extrapolated to patients with “valvular AF”. Fourth, the precision of the disease diagnoses based on ICD-9-CM may be a concern. Fifth, although we have taken “advanced CKD” as a covariate in the Cox model when evaluating the adjusted risk for subsequent AF, the confounding effect of subsequent impaired renal function and chronic dialysis could not be completely excluded. Sixth, the start point of the follow-up period is indeed arguable. Some confounding effect from acute dialysis may exist, which probably increases the risk of AF in “AKI-recovery group”, if immediate period after discharge is included in the observation period. Nevertheless, changing the start point from “after discharge” to “the 31st day after discharge” decreases 11% AF events without changing the results of the current study.

However, despite widespread interest and extensive research on AF, our understanding of the etiology and pathogenesis of this disease process is still incomplete. As a result, there are no set primary preventive strategies in a place apart from general cardiac risk factor prevention goals. Our result seems intuitive that a better understanding of acute dialysis as the risk factors for AF would better prepare medical professionals to initially prevent or subsequently treat these patients and follow up with groups who could not wean from acute dialysis.

## 5. Conclusions

In this current nationwide cohort study, we found that the experience of severe AKI necessitating dialysis carries an increased risk of subsequent AF, even in those weaned from acute dialysis. Further study is needed to determine the mechanisms which link AKI and subsequent AF and to identify potentially modifiable risk factors to decrease the burden of AF and subsequent risk of major adverse events.

## Figures and Tables

**Table 1:** Comparisons of the baseline characteristics among the three groups.

	Before Matching *n* = 20,540				After Matching *n* = 11,680			
Variables	Non-Recovery *n* = 4807	AKI-Recovery *n* = 5283	Non-AKI *n* = 10,450	*p*	Non-Recovery *n* = 2895	AKI-Recovery *n* = 2895	Non-AKI *n* = 5790	*p*
Age, years	59.7 ± 15.2	60.3 ± 17.7	59.8 ± 16.1	<0.001	59.6 ± 15.7	60.2 ± 17.2	60.6 ± 15.7	0.01
Gender, men	2390 (49.7%)	3172 (60.0%)	5540 (53.0%)	<0.001	1542 (53.3%)	1535 (53.0%)	3178 (54.9%)	0.68
**Monthly income, NTD**				<0.001				0.21
<19,100	1700 (35.4%)	2103 (39.8%)	3649 (34.9%)		1064 (36.8%)	1099 (38.0%)	2203 (38.1%)	
19,100–41,999	2449 (51.0%)	2434 (46.1%)	5285 (50.6%)		1443 (49.8%)	1378 (47.6%)	2925 (50.5%)	
≥42,000	658 (13.7%)	746 (14.1%)	1516 (14.5%)		388 (13.4%)	418 (14.4%)	762 (13.2%)	
Hospital level *				<0.001				<0.001
Level 1	1916 (39.9%)	2648 (50.1%)	4046 (38.7%)		1218 (42.1%)	1184 (40.9%)	2641 (45.6%)	
Level 2	2092 (43.5%)	2158 (40.84%)	3972 (38.0%)		1249 (43.1%)	1340 (46.3%)	2282 (39.4%)	
Levels 3 + 4	799 (16.6%)	477 (9.0%)	2,432 (23.3%)		428 (14.8%)	371 (12.8%)	967 (16.7%)	
**Baseline Comorbidities**								
CCI	2.8 ± 1.6	2.1 ± 1.5	2.4 ± 1.6	<0.001	2.4 ± 1.6	2.4 ± 1.6	2.4 ± 1.6	0.29
Myocardial infarction	22 (0.5%)	27 (0.5%)	37 (0.4%)	0.31	12 (0.4%)	10 (0.4%)	22 (0.4%)	0.91
Congestive heart failure	287 (6.0%)	215 (4.1%)	457 (4.4%)	<0.001	131 (4.5%)	136 (4.7%)	279 (4.8%)	0.91
Peripheral vascular disease	26 (0.5%)	34 (0.6%)	54 (0.5%)	0.59	13 (0.5%)	17 (0.6%)	39 (0.7%)	0.47
Cerebrovascular disease	284 (5.9%)	335 (6.3%)	629 (6.0%)	0.62	175 (6.0%)	173 (6.0%)	345 (6.0%)	0.94
Dementia	24 (0.5%)	74 (1.4%)	68 (0.7%)	<0.001	20 (0.7%)	19 (0.7%)	35 (0.6%)	0.85
COPD	333 (6.9%)	362 (6.9%)	640 (6.1%)	0.08	179 (6.2%)	209 (7.2%)	414 (7.2%)	0.23
Rheumatologic disease	47 (1.0%)	61 (1.2%)	103 (1.0%)	0.57	30 (1.0%)	28 (1.0%)	69 (1.2%)	0.66
Peptic ulcer disease	403 (8.4%)	350 (6.6%)	691 (6.6%)	<0.001	205 (7.1%)	207 (7.2%)	450 (7.8%)	0.55
Hemiplegia or paraplegia	10 (0.2%)	20 (0.4%)	25 (0.2%)	0.18	8 (0.3%)	9 (0.3%)	13 (0.2%)	0.71
CKD	2396 (49.8%)	1063 (20.1%)	3225 (30.9%)	<0.001	1005 (34.7%)	932 (32.2%)	1910 (33.0%)	0.06
Liver disease ^a^	178 (3.7%)	204 (3.9%)	355 (3.4%)	0.30	96 (3.3%)	99 (3.4%)	192 (3.3%)	0.93
Tumor	86 (1.8%)	137 (2.6%)	195 (1.9%)	0.004	62 (2.1%)	56 (1.9%)	113 (2.0%)	0.77
Diabetes	1254 (26.1%)	1186 (22.5%)	2653 (25.4%)	<0.001	687 (23.7%)	708 (24.5%)	1504 (26.0%)	0.16
**CHA2DS2-VASc †**				<0.001				<0.001
0	211 (4.4%)	706 (13.4%)	1018 (9.7%)		204 (7.1%)	200 (6.9%)	400 (6.9%)	
1	890 (18.5%)	996 (18.9%)	1979 (18.9%)		636 (22.0%)	631 (21.8%)	1125 (19.4%)	
2	1268 (26.4%)	976 (18.5%)	1908 (18.3%)		834 (28.8%)	613 (21.2%)	1144 (19.8%)	
3	885 (18.4%)	858 (16.2%)	1896 (18.1%)		534 (18.5%)	505 (17.4%)	1124 (19.4%)	
4	706 (14.7%)	683 (12.9%)	1462 (14.0%)		438 (15.1%)	410 (14.2%)	894 (15.4%)	
5	456 (9.5%)	483 (9.1%)	1123 (10.8%)		279 (9.6%)	307 (10.6%)	691 (11.9%)	
6	245 (5.1%)	350 (6.6%)	660 (6.3%)		194 (6.7%)	158 (5.5%)	306 (5.3%)	
7	120 (2.5%)	174 (3.3%)	296 (2.8%)		98 (3.4%)	91 (3.1%)	155 (2.7%)	
8	24 (0.5%)	50 (1.0%)	99 (1.0%)		23 (0.8%)	27 (0.9%)	45 (0.8%)	
9	2 (0.04%)	7 (0.1%)	9 (0.1%)		2 (0.1%)	3 (0.1%)	6 (0.1%)	

Note: Continuous variables were presented as a mean ± standard deviation, and statistically analyzed using one-way analysis of variance, while categorical variables were described as counts (percentages) and analyzed using Chi-squared tests. † denotes “before discharge”; ^a^ moderate or severe liver disease. * Level 1, 2, 3 and 4 denote medical center, regional hospital, district hospital and local medical clinic, respectively; Abbreviations: ACEI, angiotensin-converting enzyme inhibitor; ARB, angiotensin receptor blocker; CCI, Charlson Comorbidity Index; CKD, chronic kidney disease; COPD, chronic obstructive pulmonary disease; ESRD, end-stage renal disease; Gr, group; MACE, major adverse cardiac event; NSAID, non-steroid anti-inflammatory drug; NTD, new Taiwan Dollar.

**Table 2:** Comparisons of the variables within one year following hospital discharge and outcomes among the three groups.

	Before Matching *n* = 20,540				After Matching *n* = 11,680			
Variables	Non-Recovery *n* = 4807	AKI-Recovery *n* = 5283	Non-AKI *n* = 10,450	*p*	Non-Recovery *n* = 2895	AKI-Recovery *n* = 2895	Non-AKI *n* = 5790	*p*
**Outpatient visits, times ^‡^**				< 0.001				0.09
0–5 visits	4596 (95.6%)	4932 (93.3%)	9297 (89.0%)		2763 (95.4%)	2747 (94.9%)	5530 (95.5%)	
6–10 visits	121 (2.5%)	151 (2.9%)	450 (4.3%)		67 (2.3%)	74 (2.6%)	190 (3.3%)	
11–15 visits	66 (1.4%)	123 (2.3%)	479 (4.6%)		47 (1.6%)	50 (1.7%)	123 (2.1%)	
>15 visits	24 (0.5%)	77 (1.5%)	224 (2.1%)		18 (0.6%)	24 (0.8%)	47 (0.8%)	
**Medication for hypertension ^‡^**								
Alpha-Blocker	571 (11.9%)	629 (11.9%)	1000 (9.6%)	<0.001	358 (12.4%)	341 (11.8%)	698 (12.1%)	0.74
Beta-Blocker	2000 (41.6%)	1898 (35.9%)	3904 (37.4%)	<0.001	1129 (39.0%)	1143 (39.5%)	2369 (40.9%)	0.52
Calcium-Channel Blocker	3006 (62.5%)	2610 (49.4%)	4986 (47.7%)	<0.001	1614 (55.8%)	1630 (56.3%)	3360 (58.0%)	0.49
Diuretic	1707 (35.5%)	2485 (47.0%)	4168 (39.9%)	<0.001	1193 (41.2%)	1231 (42.5%)	2522 (43.6%)	0.35
ACEI/ARB	1725 (35.9%)	1791 (33.9%)	4176 (40.0%)	<0.001	1057 (36.5%)	1073 (37.1%)	2231 (38.5%)	0.44
**Other Medication ^‡^**								
Anti-diabetic drugs	1468 (30.5%)	1595 (30.2%)	3230 (30.9%)	0.57	849 (29.3%)	887 (30.6%)	1846 (31.9%)	0.16
Aspirin	411 (8.6%)	396 (7.5%)	1259 (12.1%)	<0.001	246 (8.5%)	247 (8.5%)	517 (8.9%)	0.88
Clopidogrel	143 (3.0%)	291 (5.5%)	443 (4.2%)	<0.001	104 (3.6%)	114 (3.9%)	212 (3.7%)	0.70
Ticlopidine	208 (4.33%)	117 (2.2%)	370 (3.5%)	<0.001	85 (2.9%)	86 (3.0%)	194 (3.4%)	0.57
Dipyridamole	1059 (22.0%)	792 (15.0%)	2224 (21.3%)	<0.001	538 (18.6%)	555 (19.2%)	1147 (19.8%)	0.61
Nitrate	868 (18.1%)	1050 (19.9%)	1262 (12.1%)	<0.001	501 (17.3%)	524 (18.1%)	1053 (18.2%)	0.71
Statin	519 (10.8%)	628 (11.9%)	1524 (15.6%)	<0.001	334 (11.5%)	355 (12.3%)	743 (12.8%)	0.35
Proton pump inhibitor	26 (0.5%)	57 (1.1%)	64 (0.6%)	0.001	24 (0.8%)	22 (0.8%)	42 (0.7%)	0.84
NSAID	3062 (63.7%)	2832 (53.6%)	7863 (75.2%)	<0.001	1811 (65.6%)	1833 (63.3%)	3707 (64.0%)	0.84
H2-blocker	1173 (24.4%)	1232 (23.3%)	2718 (26.0%)	<0.001	692 (23.9%)	739 (25.5%)	1535 (26.5%)	0.09
**Outcome ^‡‡^**								
Atrial fibrillation	384 (8.0%)	274 (5.2%)	759 (7.3%)	<0.001	214 (7.4%)	149 (5.2%)	383 (6.6%)	0.002
MACE	830 (17.3%)	617 (11.7%)	1449 (13.9%)	<0.001	467 (16.1%)	333 (11.5%)	772 (13.3%)	<0.001
Ischemia Stroke	1226 (25.5%)	824 (15.6%)	4195 (40.1%)	<0.001	696 (24.0%)	481 (16.6%)	2286 (39.5%)	<0.001
Hemorrhage Stroke	367 (7.6%)	229 (4.3%)	1176 (11.3%)	<0.001	222 (7.7%)	140 (4.8%)	719 (13.7%)	<0.001
All stroke	1399 (29.1%)	944 (17.9%)	4748 (45.4%)	<0.001	802 (27.7%)	555 (19.2%)	2617 (45.2%)	<0.001
Advanced CKD	988 (20.6%)	723 (13.7%)	702 (6.8%)	<0.001	822 (28.4%)	482 (16.6%)	423 (7.3%)	<0.001
Mortality	2486 (51.7%)	2204 (41.7%)	3025 (29.0%)	<0.001	1482 (51.2%)	1188 (41.0 %)	1890 (32.6%)	<0.001

Note: Continuous variables were presented as a mean ± standard deviation, and statistically analyzed using one-way analysis of variance, while categorical variables were described as counts (percentages) and analyzed using Chi-squared tests. † denotes “before discharge”; ^‡^ denotes “within one year following hospital discharge”; ^‡‡^ denotes “follow-up until death or the end of the study (31 December 2010)”; Abbreviations: ACEI, angiotensin-converting enzyme inhibitor; ARB, angiotensin receptor blocker; CKD, chronic kidney disease; COPD, chronic obstructive pulmonary disease; Gr, group; MACE, major adverse cardiac event; NSAID, non-steroid anti-inflammatory drug; NTD, new Taiwan dollar.

**Table 3:** Incidences and risks of atrial fibrillation development among the three groups.

	Atrial Fibrillation			Crude Risk	Adjusted Risk ^‡^	Adjusted Risk ^‡‡^
	Events	Person-Years	Incidence Rate ^§^	HR (95% CI)	aHR (95% CI)	aHR (95% CI)
Before propensity score-matching						
non-AKI	759	81,186.67	0.94	ref	ref	ref
AKI-recovery	274	24,007.06	1.14	1.08 (0.94–1.24)	1.16 (1.00–1.33)	1.15 (1.10–1.32)
non-recovery	384	28,726.82	1.34	1.36 *** (1.20–1.54)	1.62 *** (1.43–1.83)	1.58 *** (1.39–1.80)
After propensity score-matching						
non-AKI	383	45,562.79	0.84	ref	ref	ref
AKI-recovery	149	13,462.42	1.11	1.18 (0.98–1.43)	1.33 ** (1.10–1.61)	1.30 ** (1.07–1.58)
non-recovery	214	17,088.48	1.25	1.42 *** (1.20–1.68)	1.72 *** (1.45–2.03)	1.62 *** (1.36–1.94)

Note: Cox proportional hazard model was applied to exam the effect of dialysis-requiring AKI on subsequent atrial fibrillation development. ^§^ presented as “100 person-year”. ^‡^ Adjusted for Charlson Comorbidity Index, age, and gender. ^‡‡^ Adjusted for Charlson Comorbidity Index, age, gender, and advanced chronic kidney disease (time-varying covariate). ** denotes *p*-value ≤ 0.01; *** denotes *p*-value ≤ 0.001. Abbreviations: aHR, adjusted hazard ratio; CI, confidence interval; Gr, group; HR, hazard ratio.

**Table 4:** Incidences and risks of major adverse events between patients with and without atrial fibrillation.

	AF			Non-AF			Crude Risk	Adjusted Risk ^‡^
	Event	Person-Year	Incidence Rate ^§^	Event	Person-Year	Incidence Rate ^§^	HR (95% CI)	HR (95% CI)
Before propensity score-matching								
MACE	987	9230.59	10.69	2466	117,959.62	2.09	2.25 ** (2.03–2.49)	1.88 ** (1.70–2.09)
Hemorrhagic stroke	161	10,381.48	1.55	1611	121,543.88	1.33	1.20 * (1.02–1.41)	1.24 * (1.05–1.46)
Ischemic stroke	667	7077.34	9.42	5578	96,917.20	5.76	1.62 ** (1.49–1.75)	1.31 ** (1.21–1.42)
Total stroke	714	6867.78	10.40	6377	93,540.77	6.82	1.50 ** (1.39–1.62)	1.26 ** (1.17–1.36)
After propensity score-matching								
MACE	511	4729.33	10.80	1337	67,595.80	1.98	2.49 ** (2.17–2.86)	2.11 ** (1.83–2.43)
Hemorrhagic stroke	89	5321.74	1.67	992	68,827.18	1.44	1.20 (0.97–1.49)	1.23 (0.99–1.53)
Ischemic stroke	333	3766.11	8.84	3130	55,361.13	5.65	1.57 ** (1.40–1.75)	1.33 ** (1.19–1.49)
All Stroke	362	3654.72	9.90	3612	53,357.19	6.77	1.46 ** (1.31–1.63)	1.28 ** (1.15–1.43)

Note: Cox proportional hazard model was applied to exam the effect of dialysis-requiring AKI on subsequent atrial fibrillation development. ^§^ presented as “100 person-year”. ^‡^ Adjusted to Charlson Comorbidity Index, age, and gender. * denotes *p*-value ≤ 0.05; ** denotes *p*-value ≤ 0.001; Abbreviations: aHR, adjusted hazard ratio; CI, confidence interval; ESRD, end-stage renal disease; HR, hazard ratio; MACE, major adverse cardiac event.

## Supplementary Materials

The following are available online at http://www.mdpi.com/2077-0383/7/9/248/s1, Supplementary File 1. Validation of AKI code by NSARF; Validation of CKD code by NSARF; Definition of advanced CKD.

## References

[1] Rahman F., Kwan G.F., Benjamin E.J. (2014). Global epidemiology of atrial fibrillation. Nat. Rev. Cardiol..

[2] Chan L., Mehta S., Chauhan K., Poojary P., Patel S., Pawar S., Patel A., Correa A., Patel S., Garimella P.S. (2016). National trends and impact of acute kidney injury requiring hemodialysis in hospitalizations with atrial fibrillation. J. Am. Hear. Assoc..

[3] Wu V.C., Wu C.H., Huang T.M., Wang C.Y., Lai C.F., Shiao C.C., Chang C.H., Lin S.L., Chen Y.Y., Chen Y.M. (2014). Long-term risk of coronary events after AKI. J. Am. Soc. Nephrol..

[4] Wu V.C., Wu P.C., Wu C.H., Huang T.M., Chang C.H., Tsai P.R., Ko W.J., Chen L., Wang C.Y., Chu T.S. (2014). The impact of acute kidney injury on the long-term risk of stroke. J. Am. Heart Assoc..

[5] Shiao C.C., Wu P.C., Huang T.M., Lai T.S., Yang W.S., Wu C.H., Lai C.F., Wu V.C., Chu T.S., Wu K.D. (2015). Long-term remote organ consequences following acute kidney injury. Crit. Care.

[6] Diplaris K., Ampatzidou F., Karagounnis L., Drossos G., Vlahou A. (2016). The role of blood transfusion in the development of atrial fibrillation after coronary artery bypass grafting. Thorac. Cardiovasc. Surg..

[7] Ng R.R.G., Tan G.H.J., Liu W., Ti L.K., Chew S.T.H. (2016). The association of acute kidney injury and atrial fibrillation after cardiac surgery in an asian prospective cohort study. Medicine.

[8] Maesen B., Nijs J., Maessen J., Allessie M., Schotten U. (2011). Post-operative atrial fibrillation: A maze of mechanisms. Europace.

[9] Ommen S.R., Odell J.A., Stanton M.S. (1997). Atrial arrhythmias after cardiothoracic surgery. New Engl. J. Med..

[10] Auer J., Lamm G., Weber T., Berent R., Ng C.-K., Porodko M., Eber B. (2007). Renal function is associated with risk of atrial fibrillation after cardiac surgery. Can. J. Cardiol..

[11] Lin L.-J., Cheng M.-H., Lee C.-H., Wung D.-C., Cheng C.-L., Kao Yang Y.-H. (2008). Compliance with antithrombotic prescribing guidelines for patients with atrial fibrillation—A nationwide descriptive study in taiwan. Clin. Ther..

[12] Chao T.-F., Huang Y.-C., Liu C.-J., Chen S.-J., Wang K.-L., Lin Y.-J., Chang S.-L., Lo L.-W., Hu Y.-F., Tuan T.-C. (2014). Acute myocardial infarction in patients with atrial fibrillation with a cha2ds2-vasc score of 0 or 1: A nationwide cohort study. Hear. Rhythm..

[13] Jensen P.N., Johnson K., Floyd J., Heckbert S.R., Carnahan R., Dublin S. (2012). A systematic review of validated methods for identifying atrial fibrillation using administrative data. Pharmacoepidemiol. Drug Saf..

[14] Wang W.J., Chao C.T., Huang Y.C., Wang C.Y., Chang C.H., Huang T.M., Lai C.F., Huang H.Y., Shiao C.C., Chu T.S. (2013). The impact of acute kidney injury with temporary dialysis on the risk of fracture. J. Bone Miner. Res..

[15] Chao T.-F., Liu C.-J., Tuan T.-C., Chen T.-J., Hsieh M.-H., Lip G.Y.H., Chen S.-A. (2018). Lifetime Risks, Projected Numbers, and Adverse Outcomes in Asian Patients With Atrial Fibrillation: A Report From the Taiwan Nationwide AF Cohort Study. Chest.

[16] Chien K.-L., Su T.-C., Hsu H.-C., Chang W.-T., Chen P.-C., Chen M.-F., Lee Y.-T. (2010). Atrial fibrillation prevalence, incidence and risk of stroke and all-cause death among Chinese. Int. J. Cardiol..

[17] January C.T., Wann L.S., Alpert J.S., Calkins H., Cigarroa J.E., Cleveland J.C., Conti J.B., Ellinor P.T., Ezekowitz M.D., Field M.E. (2014). 2014 AHA/ACC/HRS guideline for the management of patients with atrial fibrillation: A report of the American College of Cardiology/American Heart Association Task Force on Practice Guidelines and the Heart Rhythm Society. J. Am. Coll. Cardiol..

[18] Ologunde R., Zhao H., Lu K., Ma D. (2014). Organ cross talk and remote organ damage following acute kidney injury. Int. Urol. Nephrol..

[19] Grams M.E., Rabb H. (2012). The distant organ effects of acute kidney injury. Kidney Int..

[20] Kelly K.J. (2003). Distant effects of experimental renal ischemia/reperfusion injury. J. Am. Soc. Nephrol..

[21] Bhalodia Y.S., Sheth N.R., Vaghasiya J.D., Jivani N.P. (2011). Homocysteine-dependent endothelial dysfunction induced by renal ischemia/reperfusion injury. J. Nephrol..

[22] Kelly K.J., Meehan S.M., Colvin R.B., Williams W.W., Bonventre J.V. (1999). Protection from toxicant-mediated renal injury in the rat with anti-cd54 antibody. Kidney Int..

[23] Leaf D.E., Christov M., Jüppner H., Siew E., Ikizler T.A., Bian A., Chen G., Sabbisetti V.S., Bonventre J.V., Cai X. (2016). Fibroblast growth factor 23 levels are elevated and associated with severe acute kidney injury and death following cardiac surgery. Kidney Int..

[24] Mathew J.S., Sachs M.C., Katz R., Patton K.K., Heckbert S.R., Hoofnagle A.N., Alonso A., Chonchol M., Deo R., Ix J.H. (2014). Fibroblast growth factor-23 and incident atrial fibrillation: the multi-ethnic study of atherosclerosis (mesa) and the cardiovascular health study (chs). Circulation.

[25] Sciacqua A., Perticone M., Tripepi G., Miceli S., Tassone E.J., Grillo N., Carullo G., Sesti G., Perticone F. (2014). Renal disease and left atrial remodeling predict atrial fibrillation in patients with cardiovascular risk factors. Int. J. Cardiol..

[26] Cao W., Li A., Li J., Wu C., Cui S., Zhou Z., Liu Y., Wilcox C.S., Hou F.F. (2017). Reno-cerebral reflex activates the renin-angiotensin system, promoting oxidative stress and renal damage after ischemia-reperfusion injury. Antioxid. Redox Signal.

[27] Lane K., Dixon J.J., MacPhee I.A.M., Philips B.J. (2013). Renohepatic crosstalk: Does acute kidney injury cause liver dysfunction?. Nephrol. Dial. Transplant..

[28] Roldan V., Marin F., Fernandez H., Manzano-Fernandez S., Gallego P., Valdes M., Vicente V., Lip G.Y. (2013). Renal impairment in a “real-life” cohort of anticoagulated patients with atrial fibrillation (implications for thromboembolism and bleeding). Am. J. Cardiol..

[29] Peng X.S., Meng G.W., Zhang J., Wang X.F., Zhao L.X., Wang Y.W., Zhang L.D. (2002). Electrochemical fabrication of ordered Ag2S nanowire arrays. Mater. Res. Bull..

[30] Violi F., Pastori D., Perticone F., Hiatt W.R., Sciacqua A., Basili S., Proietti M., Corazza G.R., Lip G.Y.H., Pignatelli P. (2015). Relationship between low ankle-brachial index and rapid renal function decline in patients with atrial fibrillation: A prospective multicentre cohort study. BMJ Open.

[31] Brodsky S.V., Hebert L.A. (2016). Anticoagulant-Related Nephropathy: Is an AKI Elephant Hiding in Plain View?. J. Am. Coll. Cardiol..

[32] Bohm M., Ezekowitz M.D., Connolly S.J., Eikelboom J.W., Hohnloser S.H., Reilly P.A., Schumacher H., Brueckmann M., Schirmer S.H., Kratz M.T. (2015). Changes in renal function in patients with Atrial Fibrillation: An analysis from the RE-LY Trial. J. Am. Coll. Cardiol..

[33] Chan Y.H., Yeh Y.H., See L.C., Wang C.L., Chang S.H., Lee H.F., Wu L.S., Tu H.T., Kuo C.T. (2016). Acute kidney injury in Asians with atrial fibrillation treated with dabigatran or warfarin. J. Am. Coll. Cardiol..

[34] Soliman E.Z., Safford M.M., Muntner P., Khodneva Y., Dawood F.Z., Zakai N.A., Thacker E.L., Judd S., Howard V.J., Howard G. (2014). Atrial fibrillation and the risk of myocardial infarction. JAMA Intern. Med..

[35] O’Neal W.T., Sangal K., Zhang Z.-M., Soliman E.Z. (2014). Atrial fibrillation and incident myocardial infarction in the elderly. Clin. Cardiol..

[36] Ruddox V., Sandven I., Munkhaugen J., Skattebu J., Edvardsen T., Otterstad J.E. (2017). Atrial fibrillation and the risk for myocardial infarction, all-cause mortality and heart failure: A systematic review and meta-analysis. Eur. J. Prev. Cardiol..

[37] Emdin C.A., Wong C.X., Hsiao A.J., Altman D.G., Peters S.A., Woodward M., Odutayo A.A. (2016). Atrial fibrillation as risk factor for cardiovascular disease and death in women compared with men: Systematic review and meta-analysis of cohort studies. BMJ.

[38] Pastori D., Pignatelli P., Angelico F., Farcomeni A., Del Ben M., Vicario T., Bucci T., Raparelli V., Cangemi R., Tanzilli G., Lip G.Y.H., Violi F. (2015). Incidence of myocardial infarction and vascular death in elderly patients with atrial fibrillation taking anticoagulants. Chest.

[39] Tornyos A., Kehl D., D’Ascenzo F., Komócsi A. (2016). Risk of myocardial infarction in patients with long-term non-vitamin k antagonist oral anticoagulant treatment. Prog. Cardiovasc. Dis..

[40] Lee C.J., Gerds T.A., Carlson N., Bonde A.N., Gislason G.H., Lamberts M., Olesen J.B., Pallisgaard J.L., Hansen M.L., Torp-Pedersen C. (2018). Risk of myocardial infarction in anticoagulated patients with atrial fibrillation. J. Am. Coll. Cardiol..

[41] Li P.K.T., Burdmann E.A., Mehta R.L. (2013). World kidney day 2013: Acute kidney injury—global health alert. Am. J. Kidney Dis..

[42] Liangos O., Wald R., O’Bell J.W., Price L., Pereira B.J., Jaber B.L. (2006). Epidemiology and outcomes of acute renal failure in hospitalized patients: A national survey. Clin. J. Am. Soc. Nephrol..

