# Supplementary material for: Risk of Incident Non-Valvular Atrial Fibrillation after Dialysis-Requiring Acute Kidney Injury
Source: J Clin Med. 2018 Aug 29;7(9):248. doi: 10.3390/jcm7090248 (PMC6162837; doi:10.3390/jcm7090248)
Supplement: Supplementary file 1 [file jcm-07-00248-s001.pdf]

Supplementary File 1:

Validation of the AKI Code by NSARF

The diagnosis accuracy of acute kidney disease (AKI) by ICD-9-CM was also validated using the NSARF (the National Taiwan University Study Group on Acute Renal Failure) database.

Among all 27,065 enrollees from the database, 10,675 patients fulfilled the criteria of AKI or acute dialysis by RIFLE definition [1], and 5230 were claimed with AKI diagnosis or acute dialysis procedure. The 5230 patients had 4990 true and 240 false-positive cases. Identifying AKI based on NHI data had a positive predictive value of 98.5%, and a negative predictive value of 74.0% [2].

Validation of the CKD Code by NSARF

The diagnosis accuracy of chronic kidney disease (CKD) by International Classification of Diseases, Ninth Revision, Clinical Modification (ICD-9-CM) was validated using the NSARF database, a multi-center prospectively-constructed database of AKI incidences between 2002 and 2010.

Among 27,065 enrollees from NSARF database, 1474 patients fulfilled the criteria of pre-existing CKD (eGFR ≦ 45 mL/min/1.73 m^2) [3] by CKD-EPI (Chronic Kidney Disease Epidemiology Collaboration) definition, and 1382 were claimed with CKD diagnosis by National Health Insurance (NHI) [4]. The 1382 patients had 1206 true and 176 false-positive cases. Analysis of performance of identifying CKD based on NHI data revealed a sensitivity of 81.8%, a specificity of 99.3%, a positive predictive value of 87.3%, and a negative predictive value of 99.0%.

Definition of Advanced CKD

Advanced CKD was defined as CKD patients with serum creatinine higher than 6 mg/dL and anemia receiving concomitant erythropoiesis-stimulating agents (ESA) treatment [5].

In Taiwan's National Health Insurance Research Database (NHIRD), the rate of misdiagnosing CKD may have been modest since 2004, because a CKD prevention program has made nearly all

patients diagnosed with CKD to receive the nephrology consultation. One component in the predialysis care of this program is the provision of ESAs for no copayment to those with serum creatinine higher than 6 mg/dL.

Thus, we defined CKD patients with concomitant ESAs prescription as those with "advanced CKD" [1]. More than 75% of advanced CKD patients are found to be anemic [6].

Reference

1. Bellomo R.;Ronco C.;Kellum JA.;Mehta RL.;Palevsky P. Acute renal failure - definition, outcome measures, animal models, fluid therapy and information technology needs: the Second International Consensus Conference of the Acute Dialysis Quality Initiative (ADQI) Group. *Crit Care* **2004**, 8, R204-212.
2. Wu VC.;Wu CH.;Huang TM.;Wang CY.;Lai CF.;Shiao CC.;Chang CH.;Lin SL.;Chen YY.;Chen YM *et al*. Long-term risk of coronary events after AKI. *J Am Soc Nephrol* **2014**, 25, 595-605.
3. Levey AS.;Stevens LA.;Schmid CH.;Zhang YL.;Castro AF, 3rd.;Feldman HI.;Kusek JW.;Eggers P.;Van Lente F.;Greene T *et al*. A new equation to estimate glomerular filtration rate. *Ann Intern Med* **2009**, 150, 604-612.
4. Wang WJ.;Chao CT.;Huang YC.;Wang CY.;Chang CH.;Huang TM.;Lai CF.;Huang HY.;Shiao CC.;Chu TS *et al*. The impact of acute kidney injury with temporary dialysis on the risk of fracture. *J Bone Miner Res* **2013**.
5. Wu VC.;Wu CH.;Huang TM.;Wang CY.;Lai CF.;Shiao CC.;Chang CH.;Lin SL.;Chen YY.;Chen YM *et al*. Long-term risk of coronary events after AKI. *J Am Soc Nephrol* **2014**, 25, 595-605.
